# Paraoxonase-1 Facilitates PRRSV Replication by Interacting with Viral Nonstructural Protein-9 and Inhibiting Type I Interferon Pathway

**DOI:** 10.3390/v14061203

**Published:** 2022-05-31

**Authors:** Lin Zhang, Yu Pan, Yunfei Xu, Wenli Zhang, Wenjie Ma, Yassein M. Ibrahim, Gebremeskel Mamu Werid, He Zhang, Changyou Xia, Ping Wei, Hongyan Chen, Yue Wang

**Affiliations:** 1 State Key Laboratory of Veterinary Biotechnology, Heilongjiang Provincial Key Laboratory of Laboratory Animal and Comparative Medicine, Harbin Veterinary Research Institute, Chinese Academy of Agricultural Sciences, Harbin 150069, China; zhanglin19920402@163.com (L.Z.); gudaoqiusheng37@163.com (Y.P.); xuyunfei202203@163.com (Y.X.); zwl5561@163.com (W.Z.); qd1992mwj@163.com (W.M.); yassin8322@gmail.com (Y.M.I.); ashenafymamo@gmail.com (G.M.W.); zhanghe3789@163.com (H.Z.); 2 College of Veterinary Medicine, Northeast Agricultural University, Harbin 150030, China

**Keywords:** PRRSV, paraoxonase-1, Nsp9, type I interferon pathway

## Abstract

Paraoxonase-1 (PON1), an esterase with specifically paraoxonase activity, has been proven to be involved in inflammation and infection. Porcine reproductive and respiratory syndrome virus (PRRSV) is still a major concern in pigs and causes severe economic losses to the swine industry worldwide. In this study, the role of PON1 was investigated in porcine alveolar macrophages (PAMs) during PRRSV infection. The results showed that PRRSV replication downregulated PON1, and the knockdown of PON1 significantly decreased PRRSV replication. Similarly, PON1 overexpression could enhance PRRSV replication. Interestingly, we observed that PON1 interacted with PRRSV nonstructural protein 9 (Nsp9), the RNA-dependent RNA polymerase, and the knockdown of PON1 lowered the RNA binding ability of Nsp9, suggesting that PON1 can facilitate Nsp9 function in viral replication. In addition, the knockdown of PON1 expression led to the amplification of type I interferon (IFN) genes and vice versa. In summary, our data demonstrate that PON1 facilitates PRRSV replication by interacting with Nsp9 and inhibiting the type I IFN signaling pathway. Hence, PON1 may be an additional component of the anti-PRRSV defenses.

## 1. Introduction

Paraoxonase-1 (PON1) is a highly promiscuous enzyme responsible for hydrolyzing a wide variety of substrates, including lactones, glucuronide drugs, organophosphorus pesticides, nerve gases, and lipid peroxides [1,2]. In recent years, PON1 has been proven to play a protective role in many human diseases, such as oxidative stress, atherosclerosis, neurological disorders, cardiovascular, cancer, diabetes, and aging [3,4,5,6]. Interestingly, increasing evidence suggests that PON1 activity is involved in virus infection, including influenza A virus, hepatitis C virus (HCV), hepatitis B virus (HBV), human immunodeficiency virus (HIV), SARS-CoV-2, and others [7,8,9,10,11].

Porcine reproductive and respiratory syndrome virus (PRRSV), a member of the *Arteriviridae* family within the order *Nidovirales*, is an enveloped, positive-sense single-stranded RNA virus [12,13]. The genome of PRRSV is approximately 15,000 nucleotides organized into more than 10 open reading frames (ORF)s (1a, 1b, 2a, 2b, 3, 4, 5a, 5, 6, and 7). ORF1a and 1b encode two large non-structural polyproteins (pp1a and pp1ab), which are then proteolytically processed into several smaller non-structural proteins (Nsps) [14,15]. The pp1a is cleaved into at least 10 Nsps, including four important proteases: Nsp1α (papain-like cysteine protease), Nsp1β (papain-like cysteine protease), Nsp2 (chymotrypsin-like cysteine protease), and Nsp4 (3C-like serine protease). The pp1ab is processed into Nsp9 (viral RNA-dependent RNA polymerase, RdRp), Nsp10 (RNA helicase), Nsp11 (endoribonuclease), and Nsp12 (involved in viral subgenomic mRNA synthesis) [16,17,18,19,20,21]. These Nsps have been identified to be involved in the replication and synthesis of the viral genome, and some of them may contribute to viral pathogenesis by modulating the host innate immune response [14,22].

PRRSV has a very narrow host and cellular tropism, preferentially infecting porcine alveolar macrophages (PAMs) [23,24]. The clinical symptoms are mainly characterized by respiratory distress in young swine and reproductive failure in pregnant sows [25]. However, there are no effective vaccines or antiviral drugs available for this virus [26]. Therefore, it is important to explore the immune regulatory mechanisms employed by the host against PRRSV infection. 

Despite PON1’s antioxidant, anti-inflammatory, and lipid oxidation-reducing activities [27,28,29,30], little is known about its role in virus infection. Previous research demonstrated that the porcine PON1 is highly expressed in the kidney, followed by the liver, lung. and small intestine [31]. Our previous study also showed that PON1 was highly expressed in PAMs [32]. In this study, we investigated the role of PON1 during PRRSV infection, and the results revealed the novel function of porcine PON1.

## 2. Materials and Methods

### 2.1. Cells and Viruses 

Marc-145 cells and HEK-293T cells were cultured in Dulbecco’s modified eagle medium (DMEM, Gibco, Waltham, MA, USA) supplemented with 10% heat-inactivated fetal bovine serum (FBS, Gibco, Waltham, MA, USA) at 37 °C under 5% CO2. Primary PAMs were prepared as previously described [32]. PAMs were cultured in RPMI-1640 medium (Gibco, Waltham, MA, USA), supplemented with 10% heat-inactivated FBS and 2% penicillin and streptomycin (Gibco, Waltham, MA, USA). The high pathogenic-PRRSV (HP-PRRSV) strain HuN4 (GenBank no. EF635006) with a titer of 1.0 × 10^6^ TCID_50_/mL was stored at −80 °C. 

### 2.2. Animal Infection Experiments

Animals for the experiment were approved by the Animal Ethics Committee of Harbin Veterinary Research Institute (Approval ID: 200720–01). All animal experiments were performed according to the regulations and guidelines for Animal Experimentation of the Harbin Veterinary Research Institute of the Chinese Academy of Agricultural Sciences. Ten five-week-old SPF pigs were randomly divided into two groups and infected with PRRSV as described in previous experiments [33]. In brief, five piglets were the mock infection control, and the other five piglets were infected with HP-PRRSV HuN4. Lung tissues were collected at 3 dpi and stored at −80 °C.

### 2.3. RNA Interference Assay

Three different small interfering RNAs (siRNAs) targeting the porcine *PON1* gene were designed and synthesized by Sigma (Sigma, Northbrook, IL, USA) (Table 1). The PON1 siRNAs and negative control siRNA at the concentration of 60 nM were transfected into PAMs (at ~80% confluence) for 24 h using Lipofectamine™ RNAiMAX (Invitrogen, Carlsbad, CA, USA) according to the manufacturer’s instructions. 

### 2.4. Construction of Plasmids and Transfection of Cells

The porcine *PON1* gene was amplified from the cDNA of PAMs using primers listed in Table 2. The amplicons were cloned into the pCAGGS vector (Addgene, Cambridge, MA, USA) with C-terminal-HA and pLVX-IRES-ZsGreen1 (Addgene, Cambridge, MA, USA) with a C-terminal-Flag, respectively, and sequenced. Genes of PRRSV nonstructural proteins, inserted into the pCAGGS vector containing a C-terminal Flag tag, were stored in the lab. The correct recombinant plasmids were extracted using the Endotoxin-Free Plasmid DNA Miniprep Kit (Tiangen, Beijing, China). Furthermore, the plasmids were transfected into HEK-293T cells at a final concentration of 1 μg/mL using the X-tremeGENE HP DNA transfection reagent (Roche, Indianapolis, IN, USA) according to the manufacturer’s instruction, and the empty vector pCAGGS was used as the negative control. 

### 2.5. PON1 Overexpression by Lentiviral Expression System and Activator

The lentiviral packaging system containing plasmids pLVX-IRES-ZsGreen1, psPAX2, and pVSV-G was purchased from Addgene (Cambridge, MA, USA). According to the manufacturer’s protocol, the packaging plasmids pLVX-IRES-ZsGreen1-PON1, psPAX2, and pVSV-G were mixed with the appropriate proportion (3:2:1) and co-transfected into HEK-293T cells with the X-tremeGENE HP DNA transfection reagent. The medium was replaced at 24 h after transfection. Supernatants were collected at 48 h after transfection, pooled, filtered with a 0.45-μm filter (Pall Corporation, Port Washington, NY, USA), and stored for use at −80 °C. PAMs were transduced with lentiviruses expressing the porcine PON1 for 24 h. Fenofibrate (MCE, South Brunswick, NJ, USA), an activator of PON1, was added to PAMs at concentrations of 40 μM for 24 h, and DMSO was used as a negative carrier control.

### 2.6. Virus Incubation 

PAMs were infected with PRRSV strain HuN4 at a multiplicity of infection (MOI) of 0.1, unless otherwise stated. After incubation at 37 °C for 1 h, PAMs were washed with serum-free RPMI-1640 and then further incubated in a maintenance medium containing 2% FBS for an additional 24 h or indicated time points. The cells were collected for quantitative real-time PCR (qPCR) or Western blotting analysis, and the supernatants were collected for virus titration in Marc-145 cells.

### 2.7. RNA Extraction and Quantitative PCR 

Total RNA was extracted using the Simply P total RNA extraction kit (BioFlux, Beijing, China) and then reverse transcribed to cDNA using primescript RT reagent with a gDNA eraser kit (Takara, Japan), according to the manufacturer’s instructions. The qPCR assay was carried out using specific primers listed in Table 3, as previously described [34]. The relative mRNA levels were normalized to β-actin. The data analysis used the cycle threshold (ΔΔCT) method [35]. 

### 2.8. Virus Titration

The virus titer was performed as previously described, with minor modifications [36]. Briefly, Marc-145 cells were cultured in 96-well plates at 24 h before virus inoculation. Virus supernatants were prepared by 10-fold serial dilution, and Marc-145 cells were then inoculated with 100 µL of the serial dilutions for 4–5 days. The presence of the cytopathic effect was observed daily. The 50% tissue culture infective dose (TCID_50_) was calculated using the method of Reed and Muench as previously described [37].

### 2.9. Coimmunoprecipitation (Co-IP) and Western Blotting

For the Co-IP assay, plasmids containing HA-tagged PON1 and Flag-tagged PRRSV Nsps (Nsp1α, Nsp1β, Nsp4, Nsp9, Nsp10, Nsp11, and Nsp12) were co-transfected into HEK-293T cells. The transfected cells were harvested at 48 hours post-transfection, then washed with cold PBS, and lysed with cell lysis buffer containing 1 mM PMSF (HaiGene, Harbin, China) at 4 °C for 1 h. Cell lysates were centrifuged at 12,000× *g* for 30 min, and supernatants were precleared with protein A&G-agarose (Roche, Indianapolis, IN, USA) on ice for 2 h. The samples were centrifuged at 3000× *g* for 10 min to eliminate the nonspecific proteins. The supernatants were incubated with 1 μg mouse anti-Flag mAb (Abmart, Shanghai, China) or mouse anti-HA mAb (1 μg) (Abmart, Shanghai, China) over night at 4 °C with gentle rotation. Subsequently, 30 μL of protein A&G-agarose was added to each lysate and incubated for 2 h at 4 °C. The beads were collected by centrifugation at 2500× *g* for 5 min and then washed with cold PBS for five times. The bound proteins were subjected to Western blotting analysis. 

Western blotting assays were performed as previously described [36]. In brief, cell lysates were separated by 12% SDS-PAGE gel under reducing conditions and transferred into polyvinylidene difluoride membranes (Merck Millipore, Temecula, CA, USA). The membranes were blocked in 5% skim milk and incubated with the indicated primary antibodies and appropriate secondary antibodies. The rabbit anti-Flag mAb (1:1000) and the rabbit anti-HA mAb (1:1000) were purchased from Abcam (Cambridge, UK). The mouse anti-β-actin mAb (1:3000) was from Sigma (Northbrook, IL, USA), and the mouse anti-PRRSV nucleocapsid (N) protein mAb was produced and purified in our laboratory and was diluted at 1:10,000. The IRDye 680 conjugated goat anti-mouse IgG and the IRDye 800 conjugated goat anti-rabbit IgG were from Li-Cor Biosciences (Lincoln, NE, USA) and used at 1:10,000. The membranes were scanned using the Odyssey infrared imaging system (LI-COR Biosciences, Lincoln, NE, USA).

### 2.10. Confocal Microscopy and Immunofluorescence Analysis (IFA) 

For confocal microscopy, HEK-293T cells were cultured in 35 mm plates. When the cells had grown up to 80% confluence, the HA-PON1 and Flag-Nsp9 plasmids were co-transfected into HEK-293T cells. The cells were fixed with 4% paraformaldehyde for 30 min and permeabilized with 0.1% Triton-X100 for 10 min. After blocking with 2% bovine serum albumin, the cells were incubated with rabbit anti-Flag (1:1000) or mouse anti-HA (1:1000; Abcam, Cambridge, UK) for 1 h at 37 °C. After washing with PBST, the cells were incubated with Alexa Fluor 488-conjugated goat anti-mouse IgG (1:200) and Alexa Fluor 594-conjugated goat anti-rabbit IgG (1:200) (Invitrogen, Carlsbad, CA, USA) for 1 h at 37 °C. Cells were washed with PBS and then stained with 4′,6-diamidino-2-phenylindole (DAPI) (Biosharp, Hefei, China) for 10 min at room temperature. The HEK-293T cells were examined using confocal microscopy (ZEISS, Jena, Germany).

An IFA assay was carried out according to the protocol of confocal microscopy with slight modification. Briefly, PAMs were transfected with siRNAs for 24 h. PRRSV was inoculated to the cell monolayers at 0.1 MOI. After 24 h, the PAMs were fixed and incubated with anti-PRRSV N protein mAb (1:1000). After washing, the PAMs were incubated with Alexa Fluor 488-conjugated goat anti-mouse IgG. Finally, PAMs were examined using an inverted fluorescence microscope equipped with a camera (ZEISS, Jena, Germany).

### 2.11. RNA Immunoprecipitation (RIP)

PAMs were infected with PRRSV at a MOI of 2.0 for 24 h. The cells were collected and lysed by complete RIP Lysis Buffer with RNase inhibitor (Takara, Japan) and protease inhibitor (HaiGene, Harbin, China) and then centrifugated at 12,000× *g* for 30 min at 4 ℃ to remove cell debris. The supernatants were precleared with Pierce protein A/G Magnetic Beads (Thermo, Waltham, USA) for 2 h with gentle rotation. The supernatants were mixed with indicated antibodies, including mouse anti-Flag antibody (Sigma, Northbrook, IL, USA), rabbit anti-Nsp9 antibody (a gift from Professor Jun Han), mouse IgG, or rabbit IgG (Abmart, Shanghai, China), and incubated at 4 °C for 4 h. Subsequently, prewashed Pierce protein A/G Magnetic Beads were added to each sample and incubated for 2 h at 4 °C. Immune complexes were then washed seven times with cold RIP wash buffer. Afterwards, total RNA was extracted from the immunoprecipitated complexes with a Simply P total RNA extraction kit (BioFlux, Beijing, China). The cDNA was synthesized using a primescript RT reagent with gDNA eraser kit (Takara, Japan), and the PCR analysis was performed using specific primers for the PRRSV-ORF7-CDS (Table 4).

### 2.12. Statistical Analysis

All statistical analyses were carried out using Prism 8.0.1 (GraphPad Software, Inc., San Diego, CA, USA) and MS-Excel. Differences between the experimental and control groups were tested by using a Student’s *t*-test (two-tails). Data are presented as the mean ± standard deviations (SD) from three or more independent experiments. A *p*-value of <0.05 was considered statistically significant.

## 3. Results

### 3.1. PRRSV Infection Induces PON1 Downregulation

**Figure 1:**
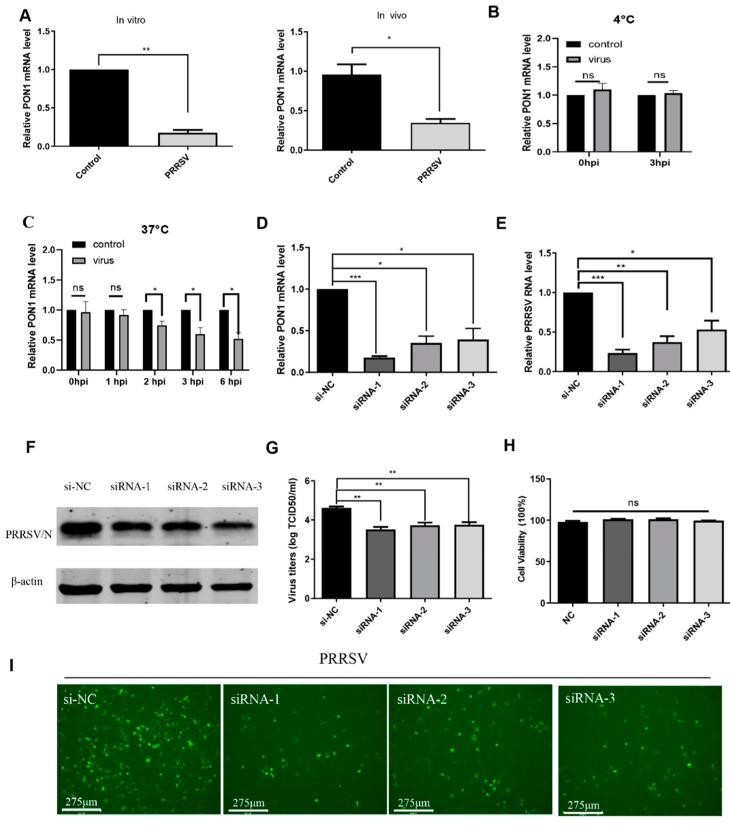
The relationship of PRRSV infection and PON1 expression. (**A**) PON1 is reduced upon PRRSV infection in vitro and in vivo. PAMs and five-week-old SPF pigs were infected with PRRSV strain HuN4. After 24 hpi of PAMs and 3 days post-infection of piglets, the RNA levels of PON1 were determined by qPCR. (**B**) Virus attachment does not affect PON1 expression. The PON1 mRNA level was determined by qPCR after incubating PAMs with the virus at 4 °C. (**C**) Downregulation of PON1 occurs after virus entry. The PON1 mRNA level was determined by qPCR after incubating PAMs with the virus for the indicated time points at 37 °C. (**D**) Three specific siRNAs of PON1 (siRNA-1, siRNA-2, and siRNA-3) and negative control siRNA (si-NC) were transfected into PAMs for 24 h, respectively. The knockdown efficiency of PON1 was determined by qPCR. (**E**–**G**) The depletion of endogenous PON1 inhibits PRRSV replication. After siRNAs were transfected into PAMs for 24 h, the PAMs were infected with PRRSV for 24 h. The effect of PON1 knockdown on PRRSV replication was determined by qPCR (**E**), Western blotting (**F**), and the TCID_50_ assay (**G**). (**H**) Transfection of siRNAs has no effect on cell viability. PAMs were transfected with three specific siRNAs of PON1 at the final concentration of 60 nM, respectively. At 24 hpi, a CCK-8 assay was performed. (**I**) PAMs were transfected with three siRNAs and the negative control for 24 h and then inoculated with the virus. At 24 hpi, the cell monolayers were examined for virus infection by IFA. Data are presented as the mean ± SD. ns (no significant difference), *p* > 0.05; *, *p* < 0.05; **, *p* < 0.01; ***, *p* < 0.001. The *p*-value was calculated using a Student’s *t*-test (two-tails).

To investigate the expression pattern of PON1 during PRRSV infection in vitro and in vivo, PAMs were incubated with PRRSV for 24 h. The qPCR results showed that the mRNA level of PON1 was significantly decreased in PRRSV-infected PAMs (left panel of Figure 1A). Additionally, the transcription level of PON1 was also determined in lungs of PRRSV-infected pigs, and Figure 1A (right panel) showed a significant decrease in PON1 mRNA expression compared to the control. Furthermore, we examined the dynamic changes of PON1 expression at different time points or under different conditions after virus inoculation. As shown in Figure 1B,C, the mRNA level of PON1 was downregulated until 2 h post-infection (hpi), suggesting that PON1 downregulation occurs after virus entry.

### 3.2. PON1 Positively Regulates PRRSV Infection

To investigate the role of PON1 in PRRSV infection, three siRNAs targeting the porcine *PON1* gene and a negative control siRNA were transfected into PAMs for 24 h. The qPCR results showed that any of the three siRNAs could effectively knock down the endogenous PON1 transcription (Figure 1D), and all three specific siRNAs resulted in a significant decrease in the PRRSV RNA level (Figure 1E) and N protein production (Figure 1F). Likewise, knockdown of the *PON1* gene by siRNAs led to a significant decrease in viral titers compared with the negative control by a TCID_50_ assay (Figure 1G). Additionally, the number of PRRSV-positive cells in PON1 knockdown groups were notably less than the control group (Figure 1I). No toxicity of siRNAs at concentrations of 60 nM were evident for PAMs (Figure 1H). Since PON1 expression can be knocked down by any of three siRNAs (siRNA-1, siRNA-2, and si-RNA-3), and the efficiency of siRNA-1 was the highest; thus, siRNA-1 was used to knock down PON1 in the following experiments.

**Figure 2:**
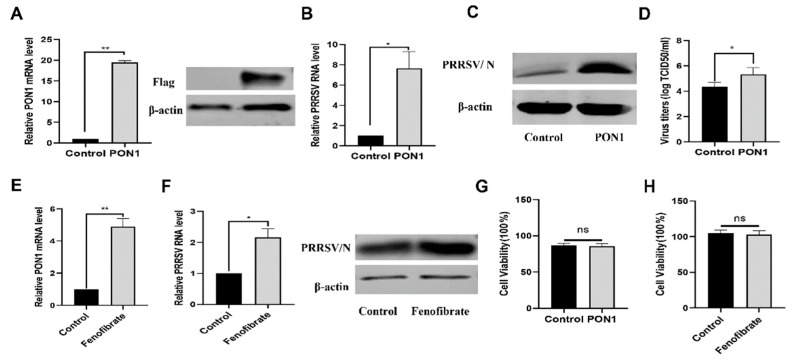
PON1 facilitates PRRSV proliferation. (**A**) Verification of PON1 overexpression. The PON1 in PAMs was mediated by lentivirus for 24 h; then, cell lysates were subjected to qPCR and Western blotting. (**B**–**D**) The overexpression of PON1 increases PRRSV replication. After the overexpression of PON1 for 24 h, PAMs were exposed to PRRSV for 24 h. The levels of viral RNA were determined by qPCR (**B**), viral protein levels were determined by Western blotting (**C**), and virus titers were detected by TCID_50_ (**D**). (**E**) PON1 is activated by fenofibrate in PAMs. PAMs were treated with 40 μM fenofibrate for 24 h, and the mRNA levels of PON1 were determined by qPCR. (**F**) Fenofibrate facilitates PRRSV proliferation. After being treated with 40 μM fenofibrate for 24 h, PAMs were exposed to PRRSV for 24 h. The levels of viral RNA were determined by qPCR, the levels of viral protein were determined by Western blotting (**F**). (**G**,**H**) In PAMs, no toxicity was observed from overexpressed PON1 and fenofibrate. PAMs were treated with either lentivirus-mediated PON1 overexpression or 40 μM fenofibrate. After 24 h, a CCK-8 assay was performed. Data are presented as the mean ± SD. ns, *p* > 0.05; *, *p* < 0.05; **, *p* < 0.01. The *p* value was calculated using a Student’s *t*-test (two-tails).

To further determine the effect of PON1 on PRRSV infection, PON1 was overexpressed with the lentivirus-mediated system (Figure 2A). As shown in Figure 2B–D, overexpressed PON1 enhanced the production of the viral RNA, N protein, and viral titer in comparison to the negative groups. To confirm these results, fenofibrate, a PON1 activator [33], was used to treat PAMs for 24 h to activate PON1 expression. The PAMs were then infected with PRRSV and incubated for an additional 24 h. The qPCR results showed that fenofibrate treatment resulted in the increase of PON1 mRNA expression (Figure 2E), viral RNA, and viral N protein when compared to the negative control (Figure 2F,G). For PAMs, no toxicity of overexpressed PON1 and fenofibrate was observed at the indicated concentrations (Figure 2G,H).

### 3.3. PON1 Regulates PRRSV Infection at the Stage of Replication

**Figure 3:**
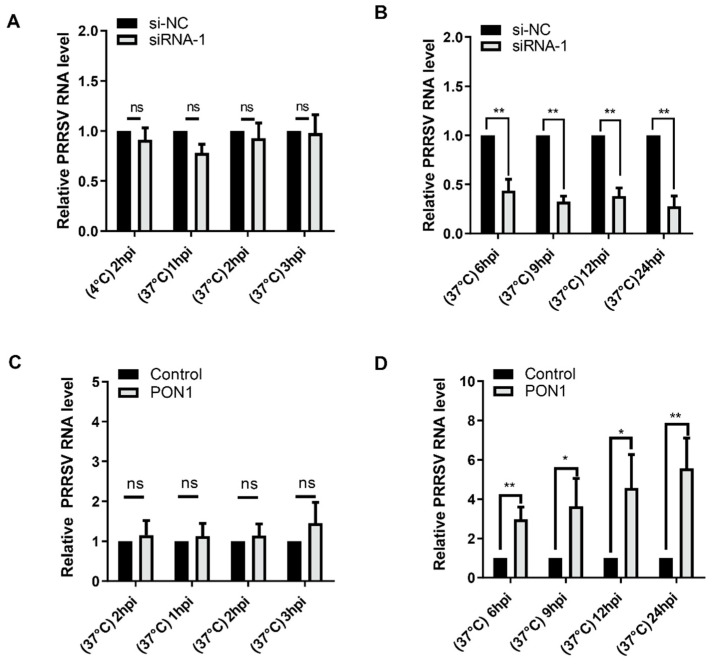
PON1 facilitates PRRSV proliferation at the stage of replication. (**A**) The depletion of endogenous PON1 does not affect virus attachment and entry. PAMs were transfected with siRNA-1 for 24 h, incubated with PRRSV at 4 °C for 2 h, and then incubated at 37 °C for 1 h to 3 h; then, virus RNA levels were determined by qPCR. (**B**) The depletion of endogenous PON1 decreases PRRSV replication. After PON1 was knocked down, PAMs were infected with PRRSV at 37 °C for 6 to 24 h. (**C**) Overexpression of PON1 does not affect virus attachment and entry. PAMs were overexpressed PON1 for 24 h and then infected with PRRSV at 4 °C for 2 h and 37 °C for 1 to 3 h.The levels of viral RNA were determined by qPCR. (**D**) The overexpression of PON1 increases PRRSV replication. After the overexpression of PON1, the PAMs were exposed to PRRSV incubated at 37 °C for 6 h to 24 h. The virus RNA levels were determined by qPCR. Data are presented as the mean ± SD. ns, *p* > 0.05; *, *p* < 0.05; **, *p* < 0.01. The *p* value was calculated using a Student’s *t*-test (two-tails).

To explore the stage at which PON1 plays an important role in the viral life cycle, PAMs were transfected with PON1-specific siRNA-1 for 24 h and then incubated with PRRSV for 2 h at 4 °C, allowing for virus attachment. Afterwards, fresh media were replaced to remove unbound virus particles, and cells were subsequently switched to 37 °C and incubated for different time points. The cell monolayers were then harvested for the detection of the viral RNA level. As shown in Figure 3A, the levels of PRRSV RNA were not affected at 2 hpi (4 °C) and at 1 to 3 hpi (37 °C), suggesting that virus attachment or penetration was not affected when the endogenous PON1 was knocked down. Conversely, we observed that the levels of PRRSV RNA were significantly decreased at 6 hpi (Figure 3B), suggesting that PON1 regulates PRRSV infection at the stage of viral biosynthesis after the virus enters the host cell. Similarly, the overexpression of PON1 did not affect virus attachment or penetration (Figure 3C), but it affected virus replication (Figure 3D). 

### 3.4. PON1 Interacts with PRRSV Nsp9 

**Figure 4:**
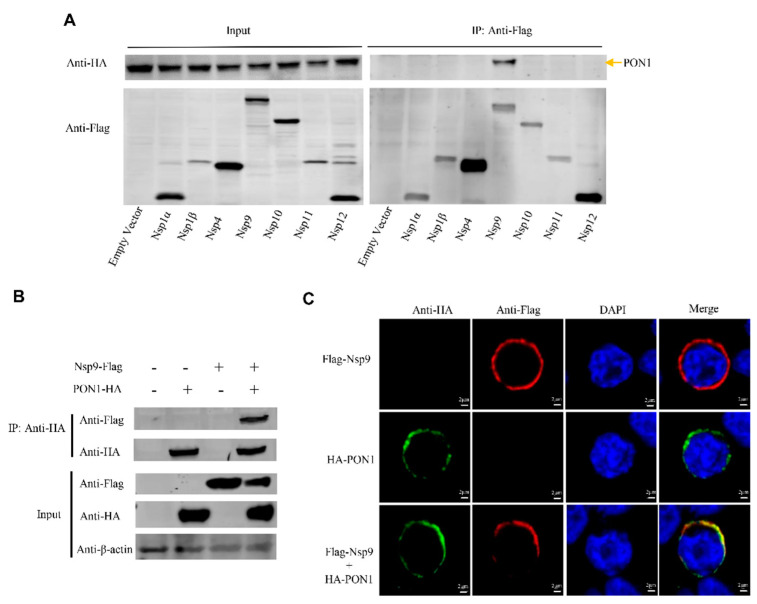
PON1 interacts with the Nsp9 of PRRSV. (**A**) Selection of the interaction of Flag-tagged PRRSV Nsps with HA-tagged PON1. (**B**) The HA-PON1 interacts with Flag-Nsp9. (**C**) Co-localization of PON1 and Nsp9. HEK-293T cells were co-transfected with HA-PON1 and Flag-Nsp9 plasmids. Cells were fixed and detected by mouse anti-HA mAb (green) and rabbit anti-Flag mAb (red). The nucleus is indicated by DAPI staining (blue).

To explore the molecular mechanism by which PON1 regulates PRRSV replication, HEK-293T cells were co-transfected with plasmids encoding the HA-tagged porcine PON1 and each Flag-tagged PRRSV Nsp (Nsp1α, Nsp1β, Nsp4, Nsp9, Nsp10, Nsp11, and Nsp12) for 48 h. Cell lysates were performed by a Co-IP assay with anti-Flag mAb and detected by Western blotting with anti-HA mAb. As shown in Figure 4A, all the exogenous proteins were efficiently expressed after transfection, and porcine PON1 was pulled down by viral Nsp9 but not by the other viral Nsps (nsp1α, nsp1β, nsp4, nsp10, nsp11, and nsp12), suggesting that Nsp9 can interact with PON1. 

To confirm the interaction between PON1 and Nsp9, HEK-293T cells were co-transfected with plasmids expressing Flag-Nsp9 and HA-PON1. Cell lysates were coimmunoprecipitated with anti-HA mAb and blotted with anti-Flag mAb. As shown in Figure 4B, Flag-tagged Nsp9 was effectively coimmunoprecipitated with HA-tagged PON1. To further confirm the interaction, HEK-293T cells were transfected with plasmids expressing Flag-Nsp9 and HA-PON1, and a confocal microscopy assay was performed with mouse anti-Flag mAb and rabbit anti-HA mAb. The confocal microscopy data illustrated that porcine PON1 and viral Nsp9 co-localized in the cytoplasm (Figure 4C), suggesting the direct interaction between PON1 and PRRSV Nsp9. 

### 3.5. The Interaction of PON1 with Nsp9 Assists PRRSV Replication

**Figure 5:**
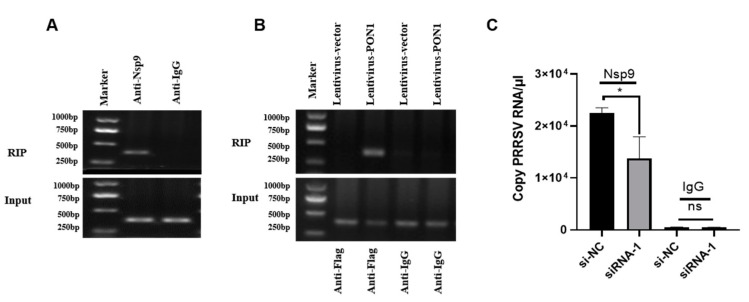
PON1 regulates the interaction of PRRSV Nsp9 with viral RNA. (**A**) Nsp9 binds to PRRSV RNA. PAMs were infected with PRRSV for 24 h, and cell lysates were subjected to RIP with an anti-Nsp9 pAb. The control IgG was included as a negative control. The RNA pulled down by the immunocomplexes was checked by RT-PCR. (**B**) The relationship between PON1 and viral RNA. Flag-PON1 was overexpressed by the lentivirus-mediated system in PAMs for 24 h; then, PAMs were infected with PRRSV for 24 h. The cell lysates were subjected to RIP with an anti-Flag mAb. The isotype control IgG was included as a negative control. The RNA pulled down by the immunocomplexes was check by RT-PCR. (**C**) Depletion of endogenous PON1 decreases Nsp9 binding to viral RNA. PON1 was knocked-down by siRNA-1; then, the PAMs infected with PRRSV were collected at 6 hpi and subjected to RIP with an anti-Nsp9 pAb or control (IgG). The virus RNA level was determined by qPCR. Data are presented as the mean ± SD. ns, *p* > 0.05; *, *p* < 0.05; The p-value was calculated using Student’s *t*-test (two-tails).

To identify the interaction between Nsp9 and viral RNA, PRRSV-infected PAMs were collected at 24 hpi and subjected to immunoprecipitation with anti-Nsp9 pAb and RNA extraction. The RT-PCR product for the PRRSV ORF7 gene was only visible in the PRRSV-infected PAMs immunoprecipitated by the anti-Nsp9 antibody but not by the control (Figure 5A). To determine the relationship between PON1 and viral RNA, Flag-PON1-overexpressed PAMs were infected with PRRSV and subjected to RIP with an anti-Flag mAb. As shown in Figure 5B, the anti-Flag mAb, but not the isotype control IgG, immunoprecipitated viral RNA from PRRSV-infected PAMs, as determined by RT-PCR, suggesting the interaction between PON1 and viral RNA. To further determine the effect of PON1 on viral replication, the endogenous expression of PON1 in PAMs was knocked down by siRNA1, and cells were infected with PRRSV for 6 h and then subjected to RIP with anti-Nsp9 pAb. The immunoprecipitated complexes were examined for the levels of viral RNA. The results showed that viral RNA immunoprecipitated by anti-Nsp9 pAb was significantly reduced in PON1-knockdown cells (Figure 5C).

### 3.6. PON1 Negatively Regulates the Type I IFN Signaling Pathway

**Figure 6:**
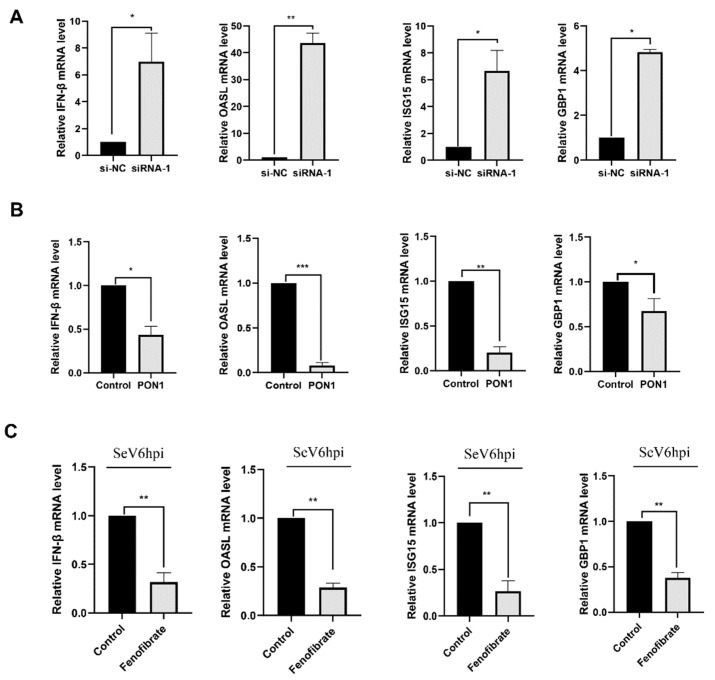
PON1 negatively regulates the type I IFN signaling pathway. (**A**) The knockdown of PON1 expression increases the expression of IFN-β and several ISGs. PAMs were transfected with PON1-specific siRNA-1 and a negative control for 24 h, and the mRNA levels of IFN-β, ISG15, OASL, and GBP1 were determined by qPCR. (**B**) PON1 overexpression results in decreased expression of IFN-β and several ISGs. Lentivirus was used to mediate the overexpression of PON1 in PAM for 24 h, and mRNA levels of IFN-β, ISG15, OASL, and GBP1 were determined by qPCR. (**C**) PON1 activator results in decreased expression of IFN-β and several ISGs. PAMs were treated with 40 μM fenofibrate for 24 h and then infected with Sendai Virus (SeV) for 6 h, and the mRNA levels of PON1 were determined by qPCR. Data are presented as the mean ± SD. *, *p* < 0.05; **, *p* < 0.01; ***, *p* < 0.001. The *p*-value was calculated using a Student’s *t*-test (two-tails).

To understand the function of PON1 on the antiviral signaling pathway, the PON1 siRNA-1-treated PAMs were collected for detecting the expression levels of IFN-β and several IFN-stimulated genes (ISG s) by qPCR. The results showed that the mRNA levels of IFN-β and several ISGs, including IFN-stimulated gene 15 (ISG15), 2′-5′-oligoadenylate synthetase-like protein (OASL), and IFN-induced guanylate-binding protein 1 (GBP1), were significantly increased when the PON1 expression was knocked down (Figure 6A). To confirm the effect of PON1 on the type I IFN signaling pathway, PON1 was overexpressed or activated by PON1 activator fenofibrate in PAMs. As shown in Figure 6B,C, the mRNA levels of IFN-β, ISG15, OASL, and GBP1 were significantly decreased in either PON1 overexpressed or fenofibrate-treated PAMs compared with the control groups. Overall, these findings suggest that PON1 negatively regulates innate antiviral responses.

## 4. Discussion

PRRSV is one of the most economically important pathogens affecting the swine industry globally [38,39,40]. To protect swine from PRRSV, vaccination has been widely implemented. However, the available PRRSV vaccines cannot provide efficient protection [41]. Therefore, the interaction between PRRSV and the host immune response needs further study.

PON1, also known as a thiolactonase, plays an. important role in cardiovascular disease and inflammatory and anti-oxidative functions in human [42,43,44]. However, little is known of PON1 in porcine, especially during virus infection. In this study, we investigated the relationship of PON1 with PRRSV infection and found that PRRSV infection reduced PON1 expression in vivo and in vitro. In addition, we demonstrated that PRRSV replication resulted in the downregulation of PON1. Though the exact mechanisms by which PON1 was downregulated by PRRSV infection are not clear, it is possible that this might happen due to the turnover of PON1 after the virus replication, or it might arise as a result of the interplay between the host immune response and the virus replication system. Virus attachment did not affect PON1 expression. Conversely, the downregulation of PON1 occurred after the virus entered the cell and during its biosynthesis, as early as 2 hpi, indicating that PON1 downregulation during PRRSV infection is independent of virus attachment but dependent on viral replication. In the current study, knocking down the *PON1* gene resulted in a significant reduction in viral replication, while PON1 overexpression increased viral replication, implying that PON1 may play a role in facilitating PRRSV replication. However, how PON1 regulates viral replication has not been investigated yet.

More than two-thirds of the PRRSV genome produces more than 12 Nsps, which are essential for viral RNA synthesis and modulating the antiviral host response [45,46]. In this study, to explore whether PON1 regulates PRRSV replication through interacting with viral Nsps, Nsps with known enzyme activity [16,17,18,19,20] were selected and screened. Interestingly, we observed that only viral Nsp9 could interact with porcine PON1. During the replicative life cycle of PRRSV, Nsp9 (the only RNA polymerase of PRRSV) is the catalytic component of the RNA genomic replication system, and it plays an essential role in virus infection [47,48]. Since PRRSV Nsp9 is considered to be a core component of viral replication and transcription complexes (RTCs) [49], the interaction of PON1 with Nsp9 implies that porcine PON1 might assist Nsp9 in carrying out its enzymatic function in PRRSV RNA synthesis. Other researchers have found that Annexin A2, retinoblastoma protein, and DEAD-box RNA helicase 5 can interact with viral NSP9, and they predict that these proteins positively regulate PRRSV replication [50,51,52]. Here, we provided the first evidence that PRRSV Nsp9 interacts with viral RNA. Furthermore, the RIP results indicate that PON1 can form a complex with viral NSP9-RNA, suggesting the critical role of PON1 during PRRSV genome replication. We speculate that PON1–Nsp9 interaction could positively affect viral replication by recruiting Nsp9 to viral RTCs, which then facilitates PRRSV biosynthesis via the interaction with viral RNA. All these findings indicate that PRRSV can hijack cellular proteins to facilitate Nsp9 enzymatic function and enhance virus replication.

Upon virus infection, the innate immune response is often activated, leading to the production of type I interferon (IFN) and cytokines, which is pivotal for the cellular antiviral responses [53]. The available evidence indicates that PON1 plays an important role in inflammation and cancer [3,28] and atherosclerosis [54], whereas its role in type I IFN signaling pathway regulation is not known yet. Here, we tried to uncover the relationship of porcine PON1 with the type I IFN signaling pathway. The mRNA levels of IFN-β, ISG15, OASL, and GBP1 were significantly decreased in either PON1-overexpressed or fenofibrate-treated groups, implying that PON1 may inhibit innate antiviral responses. This further demonstrates the role of the host immune response to PRRSV infection in reducing PON1 expression and regulation of the type I IFN signaling pathway. Some reports indicate that type I IFN and ISGs has antiviral activity against PRRSV infection [36,55]. These results suggest that PON1 downregulation can inhibit PRRSV replication through the type I IFN signaling pathway.

In summary, our findings indicate that porcine PON1 can facilitate PRRSV replication by interacting with viral Nsp9 and inhibit the type I IFN signaling pathway. Although the specific signaling pathway that PON1 affects IFN remains to be further elucidated, we have demonstrated that PRRSV infection results in the downregulation of PON1 and that PON1 plays an important role in the replication stage of PRRSV. In this study, we have uncovered a novel mechanism for understanding the roles of porcine PON1 in PRRSV replication.

## Figures and Tables

**Table 1:** Sequences of sense strand of siRNA against the target gene in PAM.

RNA Oligo Name	Sequence (Positive Strand) (5′-3′)
Negative Control	ACGUGACACGUUCGGAGAATT
siRNA-1	AUUAUCUUCAUCUGUGAAG
siRNA-2	UAGUAAACAGCAUAUGACC
siRNA-3	AAUCUAGAGACUUCAAUGG

**Table 2:** Primers used for plasmid construction.

Primer Name	Primer Sequence (5′-3′)
pCAGGS-PON1-CDS-F-KpnI	CGGGGTACCATGGCGAAGCTGATGGTGCT
pCAGGS-PON1-CDS-R-XhoI	CCGCTCGAGGAGCTCACAGTAAAGAGCTC
pLVX-PON1-CDS-F-XhoI	CCGCTCGAGATGGCGAAGCTGATGGTGCT
pLVX-PON1-CDS -R- BamHI	CGCGGATCCGAGCTCACAGTAAAGAGCTC

**Table 3:** Primers used for relative quantitative RT-PCR.

Primer Name	Primer Sequence (5′-3′)
PRRSV-ORF7-F	AGATCATCGCCCAACAAAAC
PRRSV-ORF7-R	GACACAATTGCCGCTCACTA
Porcine-β-actin-F	CTTCCTGGGCATGGAGTCC
Porcine-β-actin-R	GGCGCGATGATCTTGATCTTC
Porcine-PON1-F	CCATCAAACACAAACTTCTGCC
Porcine-PON1-R	CTCCCAGAATGTCAGGTAAGTG
Porcine-IFN-β-F	GCTAACAAGTGCATCCTCCAAA
Porcine-IFN-β-R	CCAGGAGCTTCTGACATGCCA
Porcine-ISG15-F	GATGCTGGGAGGCAAGGA
Porcine-ISG15-R	CAGGATGCTCAGTGGGTCTCT
Porcine-OASL-F	TCCCTGGGAAGAATGTGCAG
Porcine-OASL-R	CCCTGGCAAGAGCATAGTGT
Porcine-GBP1-F	GAAGGGTGACAACCAGAACGAC
Porcine-GBP1-R	AGGTTCCGACTTTGCCCTGATT

**Table 4:** Primers used for RIP analysis.

Primer Name	Primer Sequence (5′-3′)
PRRSV-ORF7-CDS-F	ATGCCAAATAACAACGGCAAGCAG
PRRSV-ORF7-CDS-R	TCATGCTGAGGGTGATGCTGTGG

## Data Availability

Not applicable.
